# Mutations in AKAP5 Disrupt Dendritic Signaling Complexes and Lead to Electrophysiological and Behavioral Phenotypes in Mice

**DOI:** 10.1371/journal.pone.0010325

**Published:** 2010-04-23

**Authors:** Michael Weisenhaus, Margaret L. Allen, Linghai Yang, Yuan Lu, C. Blake Nichols, Thomas Su, Johannes W. Hell, G. Stanley McKnight

**Affiliations:** 1 Department of Pharmacology, University of Washington School of Medicine, Seattle, Washington, United States of America; 2 Department of Pharmacology, University of California Davis, Davis, California, United States of America; Medical College of Georgia, United States of America

## Abstract

AKAP5 (also referred to as AKAP150 in rodents and AKAP79 in humans) is a scaffolding protein that is highly expressed in neurons and targets a variety of signaling molecules to dendritic membranes. AKAP5 interacts with PKA holoenzymes containing RIIα or RIIβ as well as calcineurin (PP2B), PKC, calmodulin, adenylyl cyclase type V/VI, L-type calcium channels, and β-adrenergic receptors. AKAP5 has also been shown to interact with members of the MAGUK family of PSD-scaffolding proteins including PSD95 and SAP97 and target signaling molecules to receptors and ion channels in the postsynaptic density (PSD). We created two lines of AKAP5 mutant mice: a knockout of AKAP5 (KO) and a mutant that lacks the PKA binding domain of AKAP5 (D36). We find that PKA is delocalized in both the hippocampus and striatum of KO and D36 mice indicating that other neural AKAPs cannot compensate for the loss of PKA binding to AKAP5. In AKAP5 mutant mice, a significant fraction of PKA becomes localized to dendritic shafts and this correlates with increased binding to microtubule associated protein-2 (MAP2). Electrophysiological and behavioral analysis demonstrated more severe deficits in both synaptic plasticity and operant learning in the D36 mice compared with the complete KO animals. Our results indicate that the targeting of calcineurin or other binding partners of AKAP5 in the absence of the balancing kinase, PKA, leads to a disruption of synaptic plasticity and results in learning and memory defects.

## Introduction

Mammalian cells have evolved a high degree of spatial and temporal control of signaling pathways. This specificity depends on the creation of intracellular microdomains containing signaling molecules tethered on scaffolding molecules. A kinase anchoring proteins (AKAPs) are PKA binding proteins that localize PKA and other signaling molecules to discrete sites in many cells including neurons. AKAP5, also referred to as AKAP150 in rodents, is an orthologue of human AKAP79 and bovine AKAP75 and is highly expressed in brain [1]. This AKAP binds RII subunits of PKA, as well as other signaling molecules including the calcium activated phosphatase PP2B/calcineurin [2], calmodulin [3], and PKC [4]. Thus, AKAP5 can allow for crosstalk between signaling molecules that are co-targeted as well as provide tight control of both phosphorylation and dephosphorylation of substrates present near the scaffold complex [5].

The physiological role of PKA targeting by AKAPs has been studied in neuronal cell culture and tissue slice by using the peptide, Ht-31, to disrupt endogenous AKAP/PKA interactions. The limitation of this method is that there are many AKAPs within a given cell, and Ht-31 nonspecifically disrupts all AKAP-PKA interactions. Genetic approaches can target specific AKAPs and we have generated AKAP5 knockout (KO) mice and AKAP5 PKA-binding mutant (D36) mice (AKAP5 accession number NP_001094941).

In neurons, AKAP5 targets PKA to dendritic compartments [6] and interacts with MAGUK proteins within the postsynaptic density (PSD) [7], [8]. More recently AKAP5 has been shown to directly interact with other neuronal receptors and ion channels located in the PSD, including the β-adrenergic receptor [9], [10], adenylate cyclases Type V/VI [11], the L-type calcium channel [12], [13], potassium channels, and acid sensing ion channels [7], [12], [14], [15], [16], [17]. AKAP5 has been shown to indirectly interact with and promote GluR1 phosphorylation on Ser845 by PKA [7] and on Ser831 by PKC [18]. GluR1 phosphorylation on these two sites correlates with synaptic plasticity and learning in mice [19].

Here we demonstrate that AKAP5 is required to localize both RIIα and RIIβ containing holoenzymes to the dendritic regions of neurons in the hippocampus and striatum. PKA is dramatically delocalized within dendrites in both the KO and D36 mice indicating that no other AKAPs are able to compensate and maintain normal PKA localization. We show that delocalized PKA in AKAP5 KO neurons can migrate to and associate with MAP2, an AKAP abundant in dendritic shafts. Our previous studies had demonstrated defects in both LTP and LTD in the D36 mutants and here we demonstrate that, despite similar PKA delocalization in KO and D36 mice, the electrophysiological phenotypes associated with the D36 mutant are not shared by the KO. This gain of function effect of the D36 mutant, which anchors AKAP5 binding partners but not PKA, correlates with an effect of the D36 mutation on learning and memory in an operant learning paradigm that requires reversal learning. We speculate that the targeting of a phosphatase (calcineurin) in the absence of a balancing kinase like PKA leads to a disruption of synaptic plasticity and results in learning and memory defects that are more severe in the D36 mice than those observed in the complete KO. Taken together, these results also argue that AKAP5 is primarily responsible for targeting PKA to dendritic spines and the PSD.

## Materials and Methods

All animal procedures were approved by the University of Washington's School of Medicine and the University of Iowa Institutional Animal Care and Use Committee, protocol number 2022-01 in accordance with the NIH Guide for the Care and Use of Laboratory Animals.

### Generation of mouse lines and genotyping

To create the KO mice a neomycin phosphotransferase (neo) cassette was inserted into BamH1 and BglII sites within the *AKAP*5 genomic sequence. This replaces most of the *AKAP*5 coding sequence with the neo cassette, which is oriented in the opposite direction from the *AKAP*5 gene. For the D36 mice, a stop codon (TAG) was engineered into the gene at the Leu710 position, which truncates the last 36 amino acids from the full-length protein. These 36 amino acids contain the PKA binding region. This was achieved by subcloning a 1.5 kb BglII/SpeI fragment containing the Leu710 codon from the targeting vector. Site-directed mutagenesis was performed with the QuickChange® kit from Stratagene® using Pfu polymerase with the overlapping oligonucleotides: 5′-CAGTATGAAACACTCTAGATAGAAACAGATCTTC-3′ and 5′-AGATGCTGTTTCTATCTAGAGTGTTTCATACTGTT-3′. The mutated fragment was sequenced to confirm the desired mutation. This engineered D36 mutation also introduces an XbaI restriction site that was used for genotyping. The D36 targeting vector contained the neo cassette flanked by loxP sites downstream from the *AKAP*5 sequence. Constructs were linearized with ClaI and electroporated into R1 ES cells [20]. Targeted ES clones were selected in G418 and identified by Southern Blot analysis using a 3′ external probe. Southern blots detected SacI (S) fragments of 10 kb in wild type, 12.4 kb in KO, and 11 kb in D36 recombinant ES cells. Positive ES cell clones were selected for injection into blastocysts to produce chimeric mice that were then bred to C57BL/6 to obtain germ line transmission. We further crossed D36 F1 offspring to a line of rosa26-Cre recombinase expressing mice [21], which removed the neomycin resistance gene leaving a single loxP site. Both the KO and D36 mouse lines were backcrossed to C57BL/6 (Charles River) for at least 8 generations before being used for the behavioral or electrophysiological studies described here.

### Fractionation and Western blots

Samples were dounce homogenized and/or sonicated 10∶1 v/w in homogenization buffer containing 250 mM sucrose, 0.1 mM EDTA, 0.5 mM EGTA, 10 mM DTT, 20 mM Tris, pH 7.6 with detergents (1% Triton X-100, 0.5% sodium deoxycholate), and protease and phosphatase inhibitors (1 µg/ml leupeptin, 3 µg/ml aprotinin, 40 µg/ml soybean trypsin inhibitor, 0.5 mM 4-(2-aminoethyl) bezenesulfonyl fluoride, 0.1 µM microcystin-LR, 0.2 mM NaF, 0.2 mM orthovanadate). The homogenate was cleared at 1,000×g for 10 min at 4°. The pellet was discarded and the supernatant was collected for western blots.

For separation of soluble and particulate fractions, the same homogenization buffer (without detergents) was used to homogenize the tissue in a dounce homogenizer. The supernatant of the 1,000×g centrifugation was collected and recentrifuged at 100,000×g for 30 minutes. The supernatant (S) was collected and the pellet was washed by resuspension in homogenization buffer without detergent, spun as above and the supernatant discarded. The pellet was then resuspended as the particulate fraction (P) in an equal volume of homogenization buffer with 1% sodium deoxycholate (DOC) and 1% Triton X-100.

For western blots, protein (20–40 µg) in 1x sample buffer (62.5 mM Tris·Cl, 2% SDS, 5% glycerol, 0.05% bromophenol blue, pH 6.8) was separated by SDS/PAGE and transferred to nitrocellulose (Schleicher & Schuell, Keene, NH) by electrophoresis. Blots were stained with Ponceau S to verify equal loading of lanes. Blots were then washed in TBST (100 mM Tris/HCl, 150 mM NaCl, .1% Tween-20, pH 7.2), blocked (5% BSA in Tris-buffered saline, 0.1% Tween-20) for 2 hours at room temperature, washed in TBST and probed (2 h room temp or overnight at 4° C) with the primary antibodies for: anti-C (a gift of S. S. Taylor, University of California, San Diego), anti-RI (Transduction Laboratories, Lexington, KY), anti-RIIβ (Biomol, Plymouth Meeting, PA), anti-RIIα, anti-AKAP5 N-19 and C-20 (Santa Cruz Biotechnology, Santa Cruz, CA). After primary antibody incubation, blots were washed in TBST and incubated with HRP conjugated secondary antibodies (GE Healthcare UK Limited, Little Chalfont Buckinghamshire, UK) diluted 1∶10000 in 5% blotto (dry milk) TBST and incubated 1–2 hrs at room temperature. Westerns were exposed to film (Midsci, St. Louis, Mo) after the ECL reaction (GE Healthcare/Amersham, Little Chalfont Buckinghamshire, UK).

### RII Overlay assay

After blocking, blots were incubated with recombinant RIIβ diluted to 7 nM or 70 nM in 5% BSA/TBST blocking buffer and incubated with the blots for 2 hrs at room temperature in the presence of either 0.15 µM Ht-31 anchoring inhibitor peptide (DLIEEAAVSRIVDAVIEQVKAAGAY) or a control peptide, Ht-31P (DLIEEAAVSR**P**VDAVIEQVKAAGAY) (Promega, Madison WI), which contains a proline that disrupts the RII binding helix. Blots were washed with TBST, and incubated in primary antibody against RIIβ for 2 hrs at room temp. Subsequent washes and secondary antibody steps are as in the western blot methods, except all overlay blocking was done in 5% BSA.

### Immunohistochemistry and Confocal Microscopy

Adult mice were anesthetized with pentobarbital and perfused with PBS (about 40 ml, until fluid is clear) followed by ice cold PBS-buffered 4% paraformaldehyde (PFA). Brains were dissected and post fixed in 4% PFA for at least one hour at 4°C, before being transferred to 25% sucrose and sunk 1–2 days at 4°C. Fixed mouse brains were frozen in OCT (Miles Inc., Elkhart, IN) at −80°C, and sectioned in a Cryostat (25 µm sections). Sections were mounted directly to coated Superfrost slides (Fisher Scientific, Waltham, MA). Sections were washed with PBS (three times, 5 minutes each) and incubated in blocking buffer (0.1% BSA, 0.1% Triton X100, 10% serum (from same species as secondary Ab) in PBS, for at least one hour (up to 4 or 5 hours) at room temperature (RT). Sections were incubated in primary antibody (anti-RIIβ: BD Transduction Laboratory, mAb, Cat. #610625, 1∶500 dilution; anti-RIIα: Santa Cruz, rabbit polyclonal, Cat. #sc-909, 1∶200 dilution; anti-AKAP5 N-19: Santa Cruz, goat polyclonal, Cat. #sc-6446, 1∶500 dilution) diluted in blocking buffer and incubated overnight at 4°C. Brain sections were washed with PBS+0.1% Triton X-100 (4–5 times, 5-minute incubations) at RT (mild shaking). Sections were incubated in secondary antibodies diluted in blocking buffer for at least one hour (up to three hours) at RT. Dilutions used were: 1∶500-1000, Alexa fluor488-goat anti-mouse IgG or Alexa fluor568-goat anti-rabbit IgG. Sections were washed in PBST, and mounted with Fluoromount (Beckman Coulter, Inc, Fullerton, CA). For RIIα+AKAP5 staining, the blocking buffer contains normal horse serum and the secondary antibodies are Alexa Fluor488-donkey anti-rabbit and Alexa Fluor568-donkey anti-goat IgG (Alexa Fluor antibodies from Invitrogen Corp, Carlsbad, CA). Confocal imaging (Leica TCS SP/MP) was conducted at the Keck Imaging Center of the University of Washington.

### Kinase assays

Kinase assays were performed as previously published [22].

### Immunoprecipitation

Hippocampal tissue was homogenized in a polytron at 1∶10 (w/v) in 1 mL of ice-cold lysis buffer (10 mM Na_2_HPO_4_, 150 mM NaCl, 5 mM EDTA, 5 mM EGTA, 5 mM NaF, pH 7.4) with the addition of 1.0% Triton X-100, 0.5% DOC, and 1X protease and phosphatase Inhibitor Cocktails, cat#P8340, #P2850, Sigma). The 1,000×g supernatant was collected, protein concentration determined by BCA Protein Assay (cat#23227, Pierce, USA) and 500 µg of protein was diluted into 1 mL of lysis buffer containing 0.1% Triton X-100 and 1X Protease inhibitor Cocktail. Antibodies were added and incubated overnight at 4°C followed by capture with 50 µL of a 50% slurry of Protein G magnetic beads (Dynabeads®, Invitrogen) for 1 h at 4°C. The beads were washed three times with lysis buffer containing 0.1% Triton X-100 and the immunoprecipitated proteins were eluted from the beads with 2X sample buffer. The eluted samples were then separated by SDS-PAGE and assayed by Western blot. HRP conjugated anti-mouse and anti-rabbit secondary antibodies were purchased from Jackson ImmunoResearch (PA, USA). The N-19 polyclonal anti-AKAP150 antibody (1∶500), and polyclonal anti-PKA-RIIα (1∶500), were from Santa Cruz Biotechnology, Inc. (CA, USA). The monoclonal anti-CaN (1∶1000) was purchased from Sigma (MO, USA). PSD-95 antibody was mouse monoclonal MA1-046 from Thermo Scientific (Waltham MA), GluR2/3 was Chemicon (now Millipore, Billerica MA) Ab#1506, and MAP2 was sc-12012 from Santa Cruz Biotechnology, Inc (CA, USA).

### Electrophysiology

Measurements of hippocampal LTP and LTD were done as previously described [23], [24]. Mice had been backcrossed onto a C57BL/6 background for at least 8 generations. For LTD, 10–14 day old male mice and their littermate controls were assayed. For LTP, 8–12 week old male mice and littermate controls were assayed.

### Water Maze

A hidden platform was submerged 1 cm in a 4-foot diameter tank and white tempera paint was used to make the water opaque. Mice were placed in the water maze in one of three random positions, with each position being the same distance from the hidden platform. Mice exhibiting “floating” behavior on day 1 were excluded from the study. The maximum time for a trial was 3 minutes. If a mouse did not find the platform at the 3-minute point, that mouse was scored at 180 seconds, and placed on the platform for a 10 second period. If a mouse located the platform before the 180-second period, the time was scored as the time they touched the platform, and the mouse was allowed to remain on the platform for 10 seconds. Each mouse had 3 trials per day and the results for each day were averaged. Spatial learning was assessed over 9 consecutive days of training. Each mouse had one probe trial 24 hours after the last training day to assess reference memory for the platform. Probe trials were two minutes long. GraphicState 2 (Coulbourn Instruments, PA) tracking and analysis software was used to calculate time spent in each quadrant. Male and female mice between the ages of 11 and 15 weeks old were used.

### Novel Object Recognition

Mice were placed in a circular arena (diameter of 4 feet with a 3 foot wall) with overhead illumination. On days 1 and 2 each mouse had a 10-minute free run to habituate to the arena. On day 3 each mouse performed 3 five minute runs in the arena with 10 min. breaks between runs as follows: The first run was a free run in the empty arena (without objects). The second run was in the arena containing two objects. The time spent at each of the objects was recorded. After this run, one of the objects was replaced with a novel object, and the third run was in the arena with this novel object and the previously explored object. The time spent at each of the objects was again recorded. The two objects were always placed opposite each other and 1 foot from the wall. Mice were recorded by video camera. GraphicState 2 (Coulbourn Instruments) tracking and analysis software was used to calculate time spent approaching objects.

### Operant Boxes

Before the operant box sessions, the mice were weighed to determine their pretrial weight. All mice were between 6–8 weeks old at the start of the food restriction. Over a period of 7 days the mice were fed a restricted diet (2.5 g–3 g daily), which slowly reduced their weight, until they were at 85% of the pretrial weight. Mice were weighed daily and maintained at this weight for the duration of the study. After mice performed in the operant boxes, they were weighed, and placed in their home cage.

Boxes contained 2 levers, one “active” and the other “inactive”, and 2 nose poke holes on the opposite wall. The food hopper was between the two levers. Food was dispensed when the “active lever” was pressed. The box was enclosed so that mice did not see or hear the experimenter. When the session was activated, there was an interior house light providing illumination inside the box. Pressing the active lever produced an auditory tone coupled with the delivery of 1 food pellet. Pressing the inactive lever caused a 15 sec inactivation of the active lever. During this time out, the interior light turned off, and pressing either lever had no effect. At the end of the time out, the interior light turned on, and lever pressing consequences (food reward or time out) were engaged. All sessions were 20 minutes long and each animal had 1 session each day. The schedule was as follows:

Day 1–12: Training. Left lever was active.

Day 13–20: Reversal. Right lever was active. These days are called days 1–8 of the reversal learning phase.

## Results

### Generation of KO and D36 mice

**Figure 1:**
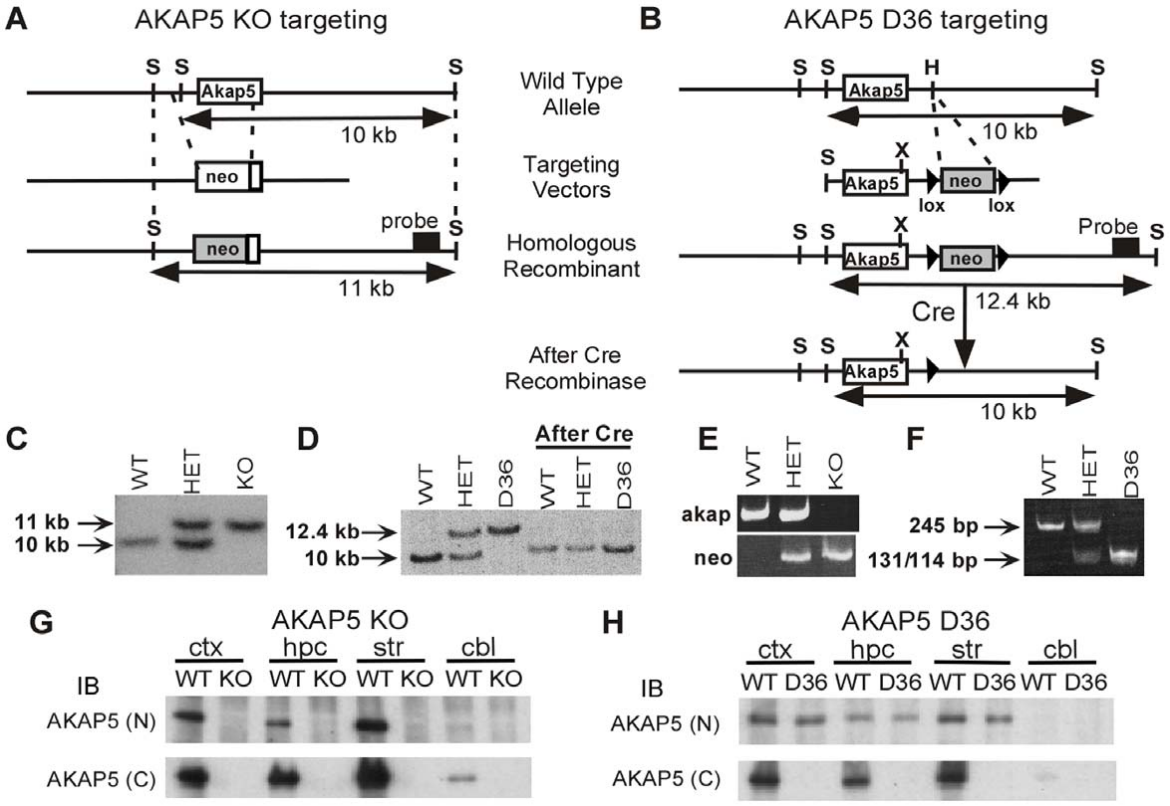
Generation of AKAP5 KO and D36 mutant mice. **A,** Targeting strategy for generating AKAP5 KO mice. The neomycin selectable marker (neo) replaces most of the coding sequence. Horizontal arrows represent the SacI (S) fragment detected in Southern Blotting. Probe represents the region hybridized to radiolabeled external probe. **B,** The targeting strategy for AKAP5 D36 mice involved insertion of a stop codon into the *Akap*5 coding sequence at position Leu710, as well as downstream insertion of the neo gene flanked by loxP sites (represented by black triangles) for subsequent removal by crossing to rosa26 cre mice. The Leu710 position is denoted with an “X” because this mutation introduced an XbaI site, which was used for genotyping, below. **C,** **D,** Southern blots confirmed the generation of the KO and D36 mice. **E,** PCR genotyping of the KO mice relies on two separate reactions. The reaction labeled “akap” amplifies sequence in the WT allele. The reaction labeled “neo” amplifies sequence in the KO allele. **F,** PCR genotyping of the D36 line of mice relies on an XbaI site created at the site of the D36 mutation. WT allele does not cut with XbaI, whereas the D36 mutant allele is cut by XbaI. **G,** **H,** Western blots in AKAP5 KO and D36 mice. AKAP5 (N) terminal antibody recognizes the N-terminus of AKAP5 that is still expressed in the D36 protein. AKAP5 (C) terminal antibody recognizes the C-terminus of AKAP5 that is lost in the D36 protein.

To study the role of AKAP5 in behavior and synaptic plasticity, we created two AKAP5 mutant lines of mice, a null allele, or knockout (KO) and an AKAP5 mutant designed to express a truncated protein missing the PKA binding domain (D36). The entire coding region of the rodent *Akap5* gene is contained within a single exon (Fig. 1A). A neomycin phosphotransferase (neo) cassette was inserted to replace most of the *Akap*5 coding sequence to generate the KO allele. Correct targeting was confirmed by Southern Blot (Fig. 1C). To create the D36 mutant allele, a stop codon was inserted into the leucine 710 position by site-directed mutagenesis (Fig. 1B). This truncates the last 36 amino acids, which contain the PKA binding portion [25]. Because the downstream *neo* gene used for selection could interfere with proper expression of the mutant protein, the floxed neo cassette was excised by crossing to the rosa26 Cre mice (Cre step in Fig. 1B) [26], and neo excision was confirmed by Southern Blot (Fig. 1D). PCR methods were devised to genotype KO and D36 mice. KO mice were genotyped with two separate PCR reactions; one reaction amplifies *Akap5* sequence contained in the WT allele, and the other amplifies *neo* sequences contained in the KO allele (Fig. 1E). In order to genotype the D36 mice, we developed a PCR strategy that takes advantage of an XbaI restriction site created by the mutation at the Leu710 position (denoted “X” in Fig. 1B). XbaI does not cut the 245 bp PCR product of the WT allele, whereas the 245 bp PCR product of the D36 allele is cut by XbaI to generate two fragments of 131 bp and 114 bp in length (Fig. 1F). Heterozygous mice have both the upper (245 bp) and lower (131/114 bp) bands.

We confirmed that the protein is absent in the KO mice (Fig. 1G), and that the mutant protein is expressed in the D36 mice (Fig. 1H). Consistent with previously published work [6], western blots demonstrate that the protein is abundant in the brain, and most abundant in the forebrain, with regions of the striatum showing the highest levels of expression whereas the cerebellum expresses at lower levels. N and C-terminal antibodies for AKAP5 demonstrate the complete absence of protein in the KO (Fig. 1G). As expected, the D36 protein is expressed but unreactive with the antibody that recognizes the deleted C-terminus of AKAP5 (Fig. 1H).

### Type II PKA is mislocalized in AKAP5 KO and D36 mice

**Figure 2:**
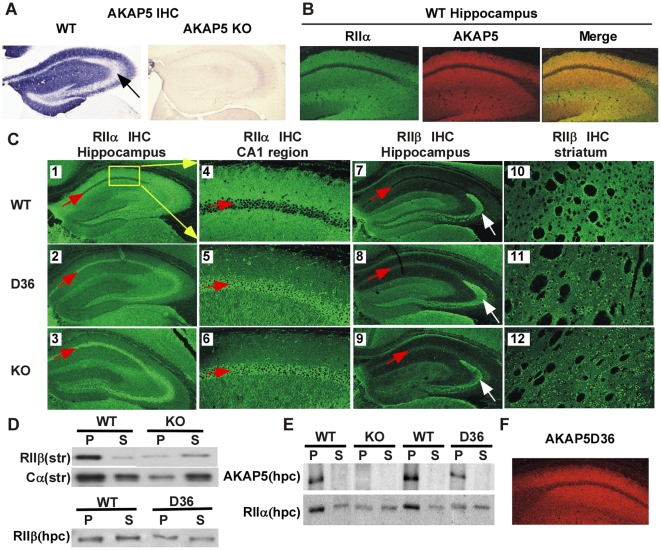
Changes in localization of signaling molecules in KO and D36 mice. **A,** Nickel enhanced diamminobenzidine (DAB) staining using an antibody against AKAP5 in the hippocampus of AKAP5 WT and KO mice. **B,** Immunohistochemistry (IHC) of RIIα, AKAP5, and the merged signal in wild type mice. **C,** IHC in whole hippocampus of RIIα in WT (panel 1), D36 (panel 2), and KO (panel 3). Higher power IHC (representing the area boxed in panel 1) of the CA1 region of RIIα in wild type (panel 4), D36 (panel 5), and KO (panel 6). IHC in whole hippocampus of RIIβ in wild type (panel 7), D36 (panel 8), and KO (panel 9). IHC in striatum of RIIβ in wild type (panel 10), D36 (panel 11), and KO (panel 12). **D,** Western blot of 100,000×g fractions (S is soluble fraction, P is particulate fraction) of RIIβ and Cα in WT and KO striatal extracts (top panels) and a comparison of S and P fractions from hippocampal tissue of WT and D36 mice (lower panel). **E,** Western blot of 100,000×g fractions of AKAP5 and RIIα in WT, KO, and D36 hippocampus (hpc). **F,** IHC in CA1 region ofAKAP5 D36 mutant protein localization in the hippocampus from a D36 mouse.

In the brain, AKAP5 is highly expressed in the dendritic regions of neurons of the forebrain. AKAP5 antibody was used in IHC to demonstrate dendritic localization in the hippocampus (Fig. 2A) and as expected the signal was lost in the KO. AKAP5 rich regions, such as hippocampal dendrites, are also abundant in RII subunits [6], [27]. AKAP5 can bind either RIIα or RIIβ subunits with nanomolar affinity [28] and, as shown in the CA1 region of WT hippocampus (Fig. 2B), RIIα (green) and AKAP5 (red) colocalized within the dendritic fields (merged yellow). RIIα and AKAP5 are localized in both the apical and basal dendrites of CA1 and CA2/3 neurons, with a paucity of staining in the cell body of these pyramidal neurons. Figure 2C panel 1 (low power) and panel 4 (higher power corresponding to the boxed area in panel 1) show the distribution of RIIα in the hippocampus of WT mice. In the D36 mice (panels 2 and 5) and KO mice (panels 3 and 6), the RIIα staining is shifted in the dendritic fields and becomes concentrated in the cell body area of the pyramidal neurons (Fig. 2, red arrows in panels 1–9 indicate the pyramidal cell layer). RIIβ is also expressed in the dendrites of CA1 and CA3 pyramidal hippocampal neurons and we observe a similar shift in the RIIβ staining (panels 8 for the D36 and 9 for the KO, red arrows). At high power this shift in RIIβ localization leads to increased RIIβ staining in the cell body and in the dendritic shafts (Figure S1). These shifts in RII localization indicate that both RIIα and RIIβ are anchored by AKAP5 in vivo and that other AKAPs are unable to completely compensate and maintain normal dendritic localization of RII subunits in these neurons.

It is known from in situ hybridization data that the granule cells of the dentate gyrus express RIIβ mRNA abundantly [29]. These neurons give rise to the mossy fiber axons that project to CA3 and RIIβ protein appears to be localized out in the presynaptic terminals of these projections (white arrows in Fig. 2C, panels 7–9). RIIβ localization in these presynaptic terminals was unaffected in the AKAP5 mutant mice and this is consistent with the observation that very little AKAP5 protein was associated with this presynaptic region (Fig. 2A, black arrow).

### RII localization by AKAP5 is not confined to the hippocampus

In the striatum, AKAP5 is highly expressed and RIIβ is the predominant regulatory subunit [30]. RIIβ was also delocalized from dendritic regions of striatal medium spiny neurons and redistributed to the cell body (Fig. 2C, panels 10–12). The PKA delocalization that we see in the hippocampus and striatum is consistent with a major role of AKAP5 in targeting RII-PKA to dendritic sites.

If AKAP5 does target PKA to the PSD, which is a highly insoluble protein-rich complex, we would expect that the amount of PKA associated with particulate fractions might be reduced in both the KO and D36 brain. AKAP5 has been shown to interact with membrane proteins at the synapse as well as scaffolds at the postsynaptic density (PSD) [7]. AKAP5, RIIα and RIIβ have all been shown to be associated with the PSD, and PSD preparations from the forebrains of AKAP5 KO mice contain 60% less RIIα and 90% less RIIβ compared to WT mice [23]. Fig. 2D shows western blots of particulate and supernatant fractions from the striatum of WT and KO mice. In the WT mice, the majority of the RIIβ and Cα are located in the 100,000×g particulate fraction (P), whereas in the KO mice the RIIβ and Cα partitioning is partially shifted into to the supernatant fraction (S) of striatal extracts. In comparison, biochemical fractionation of the hippocampus did not show a substantial shift in partitioning of the RIIβ subunit in D36 animals (Fig. 2D, bottom). This finding is consistent with the observation that the majority of the RIIβ protein in the hippocampus appeared to be localized presynaptically in mossy fiber axon terminals (white arrow, panels 7–9). This presynaptic RIIβ pool does not depend on AKAP5 for proper localization. Figure 2E shows RIIα delocalization in the hippocampus of KO and D36 mice. In the hippocampus (hpc) AKAP5 is associated with the particulate pool (top panel) and a portion of the particulate pool of RIIα seen in WT mice was shifted to the soluble fraction (S) in the KO and D36 mice (bottom panel). These shifts towards the supernatant in the mutant mice are consistent with the hypothesis that AKAP5 plays a non-redundant role in targeting PKA to the PSD in both the striatum and hippocampus. The lack of RII-PKA localization in the D36 mutant was not due to mis-folding or mislocalization of the D36 mutant itself as shown by IHC staining of D36 protein in the hippocampus (Fig. 2F), which is similar to AKAP5 staining in WT hippocampus, as seen in Fig. 2B. This agrees with previously published results demonstrating that the N-terminal domains in AKAP5 are important in membrane targeting [25]. A more central region in the AKAP5 protein that is also still expressed in the D36 protein is responsible for binding to MAGUK proteins such as PSD-95 and SAP97 located in the PSD [8].

### Compensatory changes in other AKAPs or PKA subunits are not detected in AKAP5 mutants

**Figure 3:**
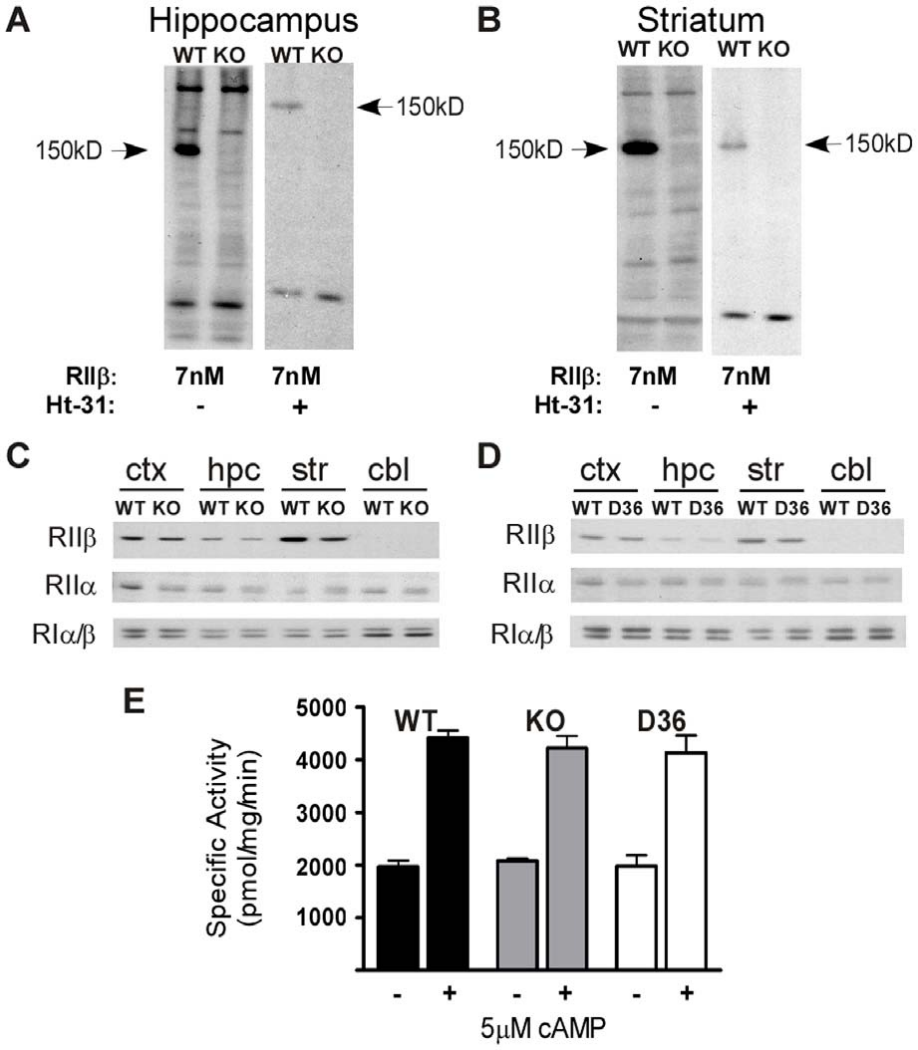
Lack of compensatory upregulation of PKA or other AKAPs in AKAP5 KO and D36 mice. **A,** RII overlay assay in WT or KO hippocampal extracts probed with RIIβ at a concentration of 7 nM with Ht-31 (+ lane) or without Ht-31 (− lanes). **B,** RII overlay assay in WT or KO striatal extracts probed with RIIβ under the same conditions as in **A**. **C,** Western blots of PKA regulatory subunits in cortex (ctx), hippocampus (hpc), striatum (str), or cerebellum (cbl) in WT and KO extracts. **D,** Western blots of PKA regulatory subunits in cortex (ctx), hippocampus (hpc), striatum (str), or cerebellum (cbl) in WT and D36 extracts. **E,** PKA activity assay representing specific activity to phosphorylate Kemptide in WT, KO, and D36 striatal extracts. Basal phosphorylation is measured in the absence (− lanes) of exogenous cAMP, and total kinase activity is measured by the addition (+ lanes) of 5 µM cAMP.

Animals surviving with a genetic mutation often develop compensatory changes that help to restore function. For example, our lab has reported that RIIβ and RIIα KO mice display up-regulation of RI subunits [31], [32]. Although the IHC results argue that AKAP5 is indispensable for PKA anchoring to dendrites, changes in expression of other AKAPs or PKA might occur in response to the AKAP5 mutations. An RII overlay assay was employed as a general approach to detect whether there are changes in other AKAPs. The results are shown for KO and WT animals in hippocampus (Fig. 3A) and striatum (Fig. 3B). The overlay assays show that AKAP5 is a major AKAP in the hippocampus. There were multiple AKAPs detected a low (7 nM) concentration of RIIβ, but there was no evidence for compensatory increases in other AKAPs in the KOs. The specificity of RIIβ binding for AKAPs was tested by including Ht-31 in the overlay incubation, which disrupts AKAP-PKA interactions. Although there are other AKAPs that could be playing a role in anchoring PKA to dendrites, these findings suggest that other AKAPs are not upregulated to compensate for the loss of AKAP5. AKAP7 (AKAP15/18) shares some of the properties of AKAP5 including membrane association, dendritic localization and ion channel interactions [33], [34] and we therefore examined its expression directly by western blot and overlay assay and found no changes in AKAP7 in WT, KO, or D36 mice (unpublished data).

Delocalization of PKA might also lead to compensatory changes in PKA-R subunit levels and western blots were performed on brain extracts from WT, KO, and D36 mice to examine this possibility. There were no changes in any of the other PKA- R subunits (Fig. 3C for KO, 3D for D36). PKA activity in the striatum of KO and D36 mice was also unaltered (Fig. 3E). Basal PKA activity (− cAMP lanes) can be indicative of free C subunits, and cAMP induced activity (+ cAMP lanes) reflects the total PKA in the tissue. From these data it appears that RII delocalization does not perturb general PKA levels or activity.

### Alterations in dendritic protein interactions in AKAP5 mutant mice

**Figure 4:**
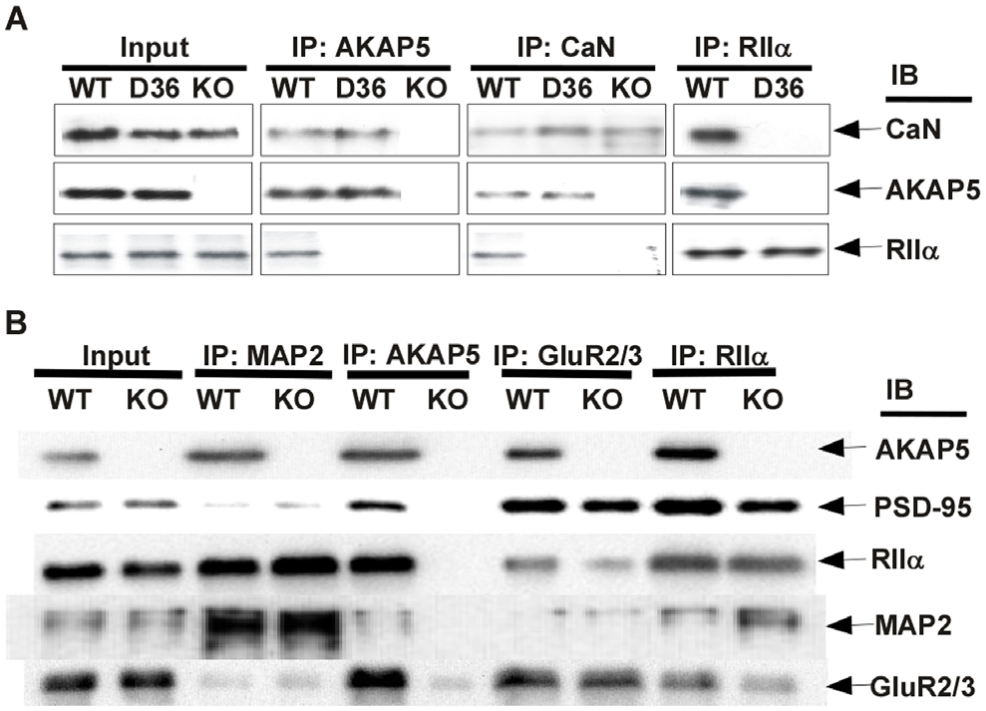
Signaling complexes in WT and AKAP5 mutant hippocampus. **A,** Immunoprecipitation (IP) of AKAP5, calcineurin (CaN), and RIIα, with immunoblotting (IB) using antibodies against CaN, AKAP5, and RIIα. Input is the equivalent amount of hippocampal extract. **B,** IP of the dendritic shaft AKAP MAP2, AKAP5, a PSD enriched protein, GluR2/3, and RIIα, with immunoblotting (IB) using antibodies against AKAP5, PSD-95, RIIα, MAP2, and GluR2/3. IP's are 7.5 fold more concentrated relative to the inputs, to facilitate visualization and comparison of the WT vs. KO extracts, as well as to detect weaker interactions.

AKAP5 protein domains that interact with other signaling molecules, such as calcineurin, have been mapped to regions still present in the D36 protein [2], [4], [35], [36], [37], [38]. Therefore we expected that the D36 protein would interact normally with these proteins in vivo even though it no longer interacts with PKA. To test this biochemically, a co-immunoprecipitation experiment was done to determine whether the D36 mutant was still able to interact with calcineurin in vivo and confirm that PKA was no longer binding (Fig. 4A). As a control we showed that immunoprecipitation of AKAP5 brought down calcineurin and RIIα as expected (Fig. 4A). Immunoprecipitation of the D36 protein brought down calcineurin but not RIIα and this was confirmed with reciprocal immunoprecipitations of calcineurin and RIIα (Fig. 4A). These results suggest that there are no other major AKAPs in hippocampus that bind both RIIα and calcineurin and confirm that the D36 AKAP5 protein can still interact with calcineurin. Together with the IHC data showing proper D36 localization (Fig. 2F), these results demonstrate that the D36 protein is targeting calcineurin, but not PKA, to AKAP5 target sites.

To more carefully characterize PKA interactions in dendrites, we performed co-immunoprecipitations of AKAP5 and PKA with markers of the PSD and the major AKAP of the dendritic shaft, MAP2. In hippocampal dendrites, RIIα is the major RII subunit anchored by AKAP5 and in the AKAP5 KO most of the RIIα moves to the cell body and dendritic shafts suggesting that it may have translocated to other AKAPs. Early biochemical studies showed that only a small percentage of MAP2 is bound by RII subunits in WT mice [39] so we tested whether this might increase in the AKAP5 KO. We found a significant increase in the association of RIIα with MAP2 in the AKAP5 KO hippocampal extracts whereas the association between RIIα and GluR2/3 was diminished (Fig. 4B). Interestingly, MAP2 showed a weak association with AKAP5 itself. These interactions could be occurring during the kinesin-mediated trafficking of PSD components along microtubules [40], or might take place in dendritic spines [41]. AKAP5 also associates with PSD-95 and GluR2/3 by immunoprecipitation but the interaction between GluR2/3 and PSD-95 is not disrupted in the AKAP5 KO. This agrees with evidence that MAGUK proteins such as SAP97 or PSD-95 can associate directly [42] or indirectly [43] with AMPA receptors. Additionally, MAGUK proteins can interact with AKAP5, which brings PKA and calcineurin into these AMPA receptor complexes [7], [8], [44].

### D36 mutant mice exhibit impaired hippocampal LTP and LTD compared with either WT or AKAP5 KO mice

**Figure 5:**
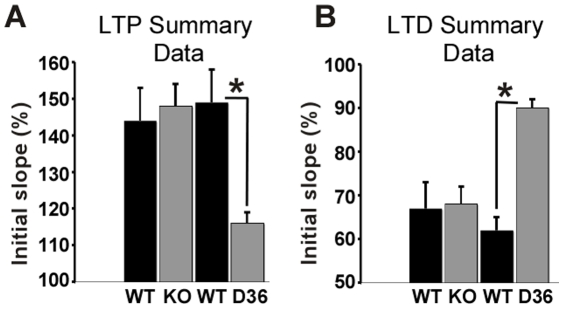
AKAP5 D36, but not KO mice, have impaired hippocampal LTP and LTD. **A,** LTP induced by stimulation of Schaffer collaterals in the CA1 area for 1 sec at 100 Hz in brain slices from KO and D36 mice compared to their littermate controls. Summary LTP comparison data from 7–12 week old KO and D36 mice and their respective littermate controls. **B,** LTD induced by stimulation of Schaffer collaterals in the CA1 area for 15 min at 1 Hz in brain slices from KO and D36 and WT control mice. Summary LTD data from 10–14 day old KO and D36 mice and their littermate controls. For each recording averages of the initial slopes measured 55–60 min after LTP or LTD induction were compared to those of the 5 min preceding LTP or LTD induction; * represents p<.05, one way ANOVA for LTP data and independently for LTD data comparing WT, KO, and D36 mice.

Pharmacologic inhibition of PKA-AKAP interactions can disrupt hippocampal plasticity [45]. We have previously shown that 8 week old D36 mice have a deficit in CA1 LTP [23]. Unexpectedly, we found that the AKAP5 KO mice did not have a detectable deficit in LTP using the same induction paradigm (Figure S2, A). Summary results of fEPSP data elicited before and after a single tetanus (1 sec/100 Hz) showed that Schaffer Collateral-CA1 (SC-CA1) LTP was deficient only in the D36 mice (Fig. 5A). These studies on the KO and D36 mice were done in parallel and therefore cannot be explained by different experimental conditions. We have also published results showing that the D36 mice showed a deficit in LTD at age 2 weeks [24]. Again, in contrast, the KO animals displayed normal LTD (Figure S2, B). The LTD data for both KO and D36 are summarized in Fig. 5B. The electrophysiological deficits in both LTP and LTD are specific to the D36 mice, and the KO mice exhibit essentially normal LTP and LTD under these stimulating conditions. We conclude that the D36 mice have an electrophysiological defect compared to KO mice of the same age.

### Spatial learning and short-term memory are intact in the D36 mice

**Figure 6:**
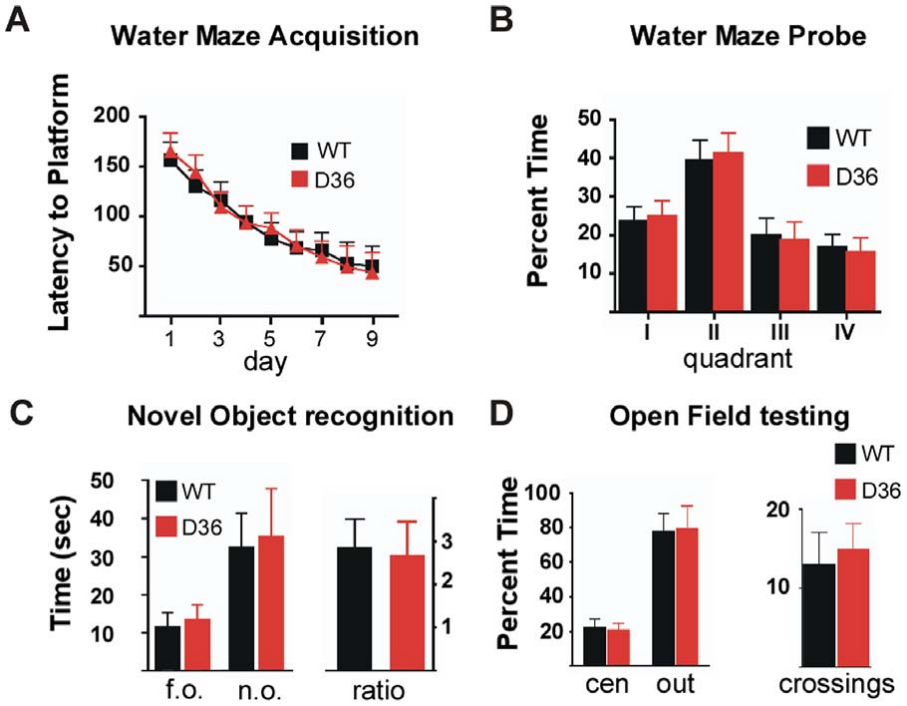
Normal spatial learning, short-term memory, and open field behaviors in AKAP5 D36 mice. **A,** Water maze learning as measured by the latency to find a hidden platform over 9 consecutive days, three trials per day, in D36 vs. WT littermate controls. Each training trial was 3 min. Data are plotted as the average of the three trials on each day. ANOVA reports a significant effect of day: F (8, 198) = 10, p<.001, but not genotype: F (1, 198) = .02, p = .88 on the latencies to the platform. **B,** Probe trial (one day after last training trial) as measured after removing the platform and counting time spent in the quadrants (quadrant II housed the platform), for D36 and littermate WT controls. Probe trial consisted of a single two-minute trial per animal on day 10, which was 24 hours after the last training day. Data are plotted as the percent time the animals spent in each quadrant. ANOVA reports only a significant effect of quadrant: F (3, 88) = 11.9, p<.05, Bonferonni post hoc test for quadrant II, and no significant effect of any other quadrant or of genotype on the probe trial: F (1, 88) = .01, p = .96 **C,** Novel object recognition as measured by the time spent in an area containing a novel object (n.o.) compared with a comparable area housing a familiar object (f.o.). Ratio is the fold increase in time spent in the area of the novel object. ANOVA analysis reports no effect of genotype on novel object preference. **D,** Open field testing measures the percent time spent toward the center of the arena (cen) vs. near the edge of the arena walls (out), with crossings equaling the number of times mice went from the edge area into the center area. Open field trials lasted 5 minutes. ANOVA analysis reports no effect of genotype on open field behavior.

Spatial learning requires intact PKA signaling in the hippocampus [46] and deficits in hippocampal LTP and LTD in mutant mice often correlate with deficits in spatial learning and memory tasks [47]. Therefore we assessed spatial learning and memory in the D36 mice by testing them in the Morris Water Maze. Both D36 and the WT littermate controls showed indistinguishable acquisition of the task as measured by latency to find the hidden platform during a 9-day training period (Fig. 6A). The probe trial was done on the 10^th^ day, and both D36 and WT mice showed an equivalent preference for the quadrant that previously housed the hidden platform (Fig. 6B). These results show that despite PKA delocalization and deficient LTP and LTD in the hippocampus, the D36 mice exhibited intact spatial learning and memory in the Morris Water Maze.

Learning and memory in the water maze task took place over nine consecutive days, after which the behavior was well entrained. We reasoned that the D36 LTP and LTD deficits might manifest in a behavior requiring short-term memories after a single trial. Novel object recognition is a test of short-term memory and has been shown to be impaired in rodents with hippocampal lesions [48]. Novel object exploratory behavior facilitates LTD in awake behaving animals at the rat SC-CA1 synapse and inhibition of LTD in this paradigm prevents habituation to novel stimuli [49]. However, the D36 mice performed this test as well as controls indicating that the D36 mice have intact short-term memory as measured by the ratio of the time spent at the novel object compared to the familiar object (Fig. 6C).

Open field testing can also be used to measure anxiety which may affect other behavioral tests. Adenylate cyclase 5 (AC5) is highly expressed in the striatum as is AKAP5 and AC5 KO mice show reductions in several anxiety like behaviors [50]. Perseverance near the edges or walls of the illuminated open field was measured and we found no differences between D36 and WT in this test (Fig. 6D) indicating that D36 mice exhibit normal exploratory behavior and do not show heightened anxiety. Despite the deficits in hippocampal LTP and LTD in the D36 mice, their spatial learning, and anxiety related behaviors remained intact. Since AKAP5 is most highly expressed in the ventral and dorsal striatum where it colocalizes with RIIβ [6] we tested whether AKAP5 mice would exhibit deficiencies similar to those seen in RIIβ KO mice. The RIIβ KO mice have increased locomotor sensitization to amphetamine, deficient rotarod performance, and are resistant to haloperidol-induced catalepsy [22], [51]. Despite the association between AKAP5 and RIIβ, the AKAP5 KO and D36 mice displayed normal behavior in all of these tests (Figure S3, and data not shown).

### Reversal learning is impaired in D36 mice but not in KO mice

**Figure 7:**
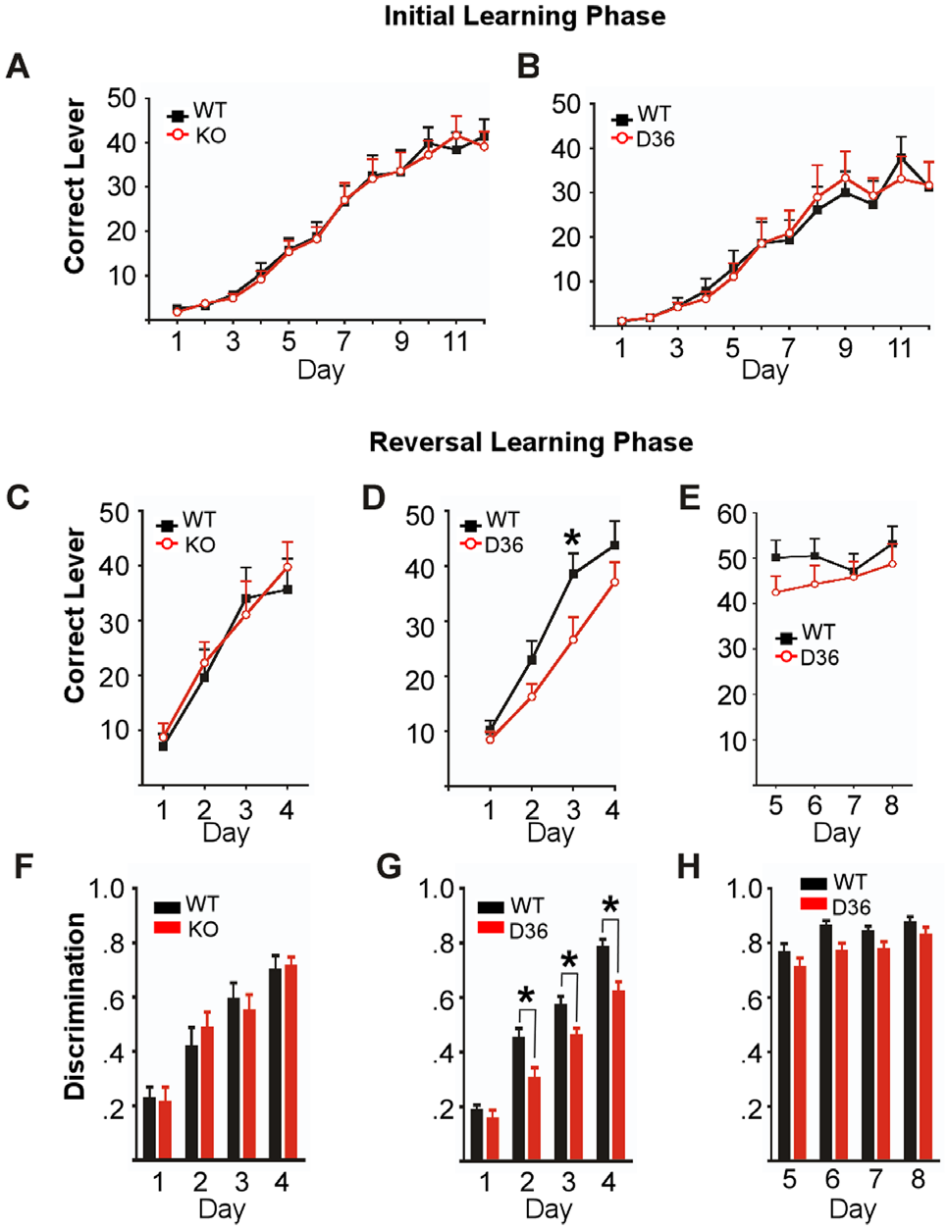
Impaired reversal learning in AKAP5 D36 but not KO mice. **A,** Initial learning phase in KO and WT littermate controls in an operant box where lever pressing for food reward is plotted. Correct lever presses are plotted for 12 consecutive days. Since the reinforcement schedule was FR = 1, this equals the number of food pellets dispensed. **B,** Correct lever presses during the initial learning phase for D36 and WT littermate controls in the same paradigm. **C,** Correct lever presses during the reversal learning phase plots performance after the active lever is switched (day 13), marking the beginning of the reversal learning phase. KO and WT littermate controls plotted during the first four consecutive days of the reversal learning. **D,** D36 and WT littermate controls plotted during the first four consecutive days of the reversal learning. **E,** Correct lever presses of the D36 and WT littermate controls, plotted for days 5–8 during the reversal learning. **F,** Discrimination represented as the fraction of correct lever presses during days 1–4 of the reversal-learning phase for KO and WT littermate controls. **G,** Discrimination during days 1–4 of the reversal-learning phase for D36 and WT littermate controls **H,** Discrimination during days 5–8 of the reversal-learning phase for D36 and WT controls. The data shown in **C–E** collected from the same trials used in calculating discrimination in **F–H**. * represents p<.05, Two Way ANOVA with Bonferroni's Post Test. **A,** **B,** For correct lever pressing during the initial learning, there is a significant effect of training day: KO group; F (11, 192) = 18.0, p<0.001, D36 group; F (11, 444) = 44.7, p<0.001, but no significant effect of genotype: KO group; F (1, 192) = .01, p = .93, D36 group; F (1, 444) = .11, p = .75. **C,** **D,** For correct lever pressing during the reversal learning, there is a significant effect of training day: KO group; F (3, 64) = 16.3, p<0.001, D36 group; F (3, 148) = 35.9, p<0.001. There is no significant effect of genotype for the KO group; F (1, 64) = .17, p = .68, but there is a significant effect of genotype in the D36 group compared to their WT littermate controls; F (1, 148) = 8.65, p = .004. **F,** **G,** For discrimination on days 1–4 of reversal learning, there is a significant effect of training day: KO group; F (3, 64) = 27.1, p<0.001, D36 group; F (3, 148) = 90.0, p<0.001. There is no significant effect of genotype for the KO group; F (1, 64) = .03, p = .87, but there is a significant effect of genotype in the D36 group compared to their WT littermate controls; F (1, 148) = 22.6, p<.05.

There are lines of mice that, like the D36 and KO mice, show deficits in hippocampal electrophysiology, yet remain within normal limits on most standard tests of memory, learning, and behavior. GluR1 mutant mice are one such example, where standard tasks like the Morris Water Maze fail to identify deficits yet certain forms of hippocampal LTP are compromised [52], [53]. However, GluR1 KO and a GluR1 mutant mouse that eliminates phosphorylation sites for PKA and PKC (GluR1 Ser845 and Ser831, respectively) exhibit behavioral deficits when faced with more complex behavioral tasks involving reversal learning [52], [54]. Therefore we challenged the KO and D36 mice to operant conditioning for food reward and reversal learning using this task. In the training phase, naive WT and AKAP5 mutant mice were each given a daily 20 min trial over 12 consecutive days to learn to press one of two levers for food. Correct lever presses are shown for WT vs. KO (Fig. 7A) and WT vs. D36 mice (Fig. 7B). Both the KO and D36 mice exhibited similar performance in the training phase of this task compared to their WT littermate controls. After this 12 day training phase, the active lever was switched such that the mice had to press a different lever to deliver the food reward. Reversal learning requires behavioral flexibility: the mice need to adapt their previously learned behavior to stop pressing the old active lever and begin pressing the new active lever for food reward. In this task the KO mice showed no deficit and learned to press the new active lever at the same rate as their WT controls (Fig. 7C). However the D36 mice showed a delayed acquisition in reversal learning relative to WT controls (Fig. 7D). This deficit was during the initial period of days where the rate of acquisition, or learning, for the new lever was at a maximum. After this initial phase (days 1–4) of reversal learning the D36 mice catch up and begin to perform the task comparable to their WT littermate controls (days 5–8, Fig. 7E). The D36 deficiency in reversal learning can also be viewed as a lack of discrimination between the active and inactive levers. Discrimination is defined as the fraction of correct lever presses, where a value of 0.5 represents equal correct vs. incorrect (random) lever pressing. Discrimination is shown for the task comparing KO (Fig. 7F) and D36 (Fig. 7G) to WT controls. As expected, discrimination by the KO mice in this task was similar to their WT controls and increased comparably over days 1–4 of the reversal learning task. However, the D36 mice were deficient as indicated by lever discrimination during the initial phase and then caught up with the WT group on days 5–8 (Fig. 7H). In the initial phase of the reversal paradigm, the discrimination for all groups of mice started well below 0.5, demonstrating that the mice were still favoring the previously active lever. This is reflected by the low number of correct lever presses during first days of reversal learning. The discrimination increased over subsequent days for all mice, but the D36 mice were deficient compared to WT littermate controls only in the rate of acquisition of the new task (days 1–4). The KO mice showed no deficit during this time of reversal learning. KO, D36 and WT mice all reached similar levels of correct lever and discriminative performance after 5 days. These data show that during the reversal-learning trial, when the task requires behavioral flexibility, D36 mice have a significant learning deficit compared with WT and more interestingly, the KO mice. This behavioral deficit correlates with the D36 deficits in hippocampal LTP and LTD and suggests that mutation of the PKA docking site on AKAP5 has created a negative effect on synaptic plasticity and behavior that is not seen in the complete AKAP5 KO. We speculate that releasing PKA but retaining the other signaling components including calcineurin may lead to this negative effect on function.

## Discussion

PKA and, more recently, AKAPs, have been shown to be important in neuronal function and plasticity. We have created two lines of AKAP5 mutant mice (KO and D36) that are informative in understanding the roles of this scaffolding molecule in intracellular signaling. KO mice lack the protein completely and therefore the entire AKAP5 signaling complex is disrupted. D36 mice express a mutant protein that is missing the PKA binding domain but continues to bind and localize other signaling molecules including the phosphatase, calcineurin. The defining feature of AKAPs is that they bind to and target PKA to specific sites within a cell and AKAP5 is one of the most abundant high affinity AKAPs in the forebrain as demonstrated by RII overlay (Fig. 3A, B). In both mutant mouse lines there is a dramatic relocalization of RIIα and RIIβ from dendritic regions to the cell bodies in the hippocampus and striatum (Fig. 2C), which is consistent with our previous findings of reduced RIIα and RIIβ in PSD complexes isolated from AKAP5 mutant mice [23]. We found no evidence for a compensatory up regulation of other AKAPs (Fig. 3) and the delocalization of PKA indicates that AKAP5 is a predominant binding partner for PKA in forebrain dendritic processes.

Despite the uncompensated PKA delocalization, these AKAP5 mutant mice are remarkably unaffected. No changes in body weight, fertility, or growth rates were evident. As one of the major binding partners of AKAP5 is the RIIβ subunit of PKA we tested whether any of the well-studied RIIβ KO phenotypes might be recapitulated in the AKAP5 KO or D36 mutants. The RIIβ KO mice are deficient in rotarod learning and haloperidol-induced catalepsy, and they are lean and hyperactive [22], [32], [51], [55]. None of these phenotypes were observed in either the AKAP5 KO or D36 mutant mouse lines (Figure S3) indicating that the phenotypes of the RIIβ KO are not likely to be caused by loss of PKA localization to AKAP5 in these animals.

Previously we reported that the D36 mice display deficits in hippocampal LTP and LTD at the SC-CA1 pathway [23], [24]. The defects in LTP were only seen in adult (8 wk) animals whereas LTP in 4 wk old mice remained intact. This age-dependent deficit was in agreement with the requirement for PKA to elicit LTP at 8 weeks but not at 4 weeks of age when using a single 100 Hz/1 sec tetanus [23] and the observations that GluR1 KO and GluR1 knockin mutants that eliminate phosphorylation sites (S831A, S845A) also show a similar age-dependent LTP deficit in adult but not young animals [56]. Examination of LTD in 2 wk old mice also demonstrated a substantial deficit in the D36 mice. However, when we examined the AKAP5 KO mice under the same conditions we found that both LTP in adult and LTD in 2 wk old mice were intact (Fig. 5). This was an unexpected result and implies that the PKA binding site mutation in the D36 animals is causing a gain of function effect on synaptic plasticity which is not seen when the protein is eliminated in the KO.

As electrophysiological deficits often correlate with behavioral deficits, we examined whether the D36 mice might have more pronounced behavioral defects compared with KO mice. The D36 animals did not appear to have deficits in simple learning tasks including novel object recognition or Morris Water Maze (Fig. 6), so we tested them in a more complex operant conditioning paradigm that required reversal learning. Mice were trained to press one of two levers for a food reward and the acquisition of this task was similar for WT, KO and D36 animals. However, when the reward lever was reversed, the D36 mice were deficient in their ability to relearn the task compared with WT and KO mice (Fig. 7). This behavioral deficit, specific to the D36 mice, correlates with their deficiency in both hippocampal LTP and LTD when compared with either WT or KO mice.

**Figure 8:**
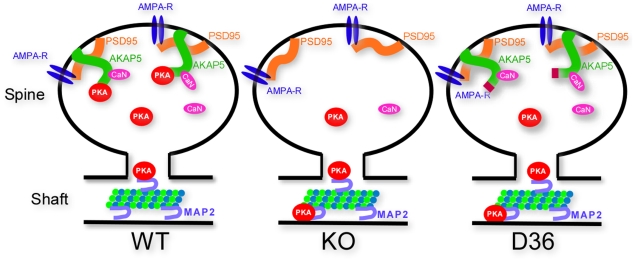
Model of the AKAP5 scaffold domain at the PSD in WT, AKAP5 KO, and AKAP5 D36 mutant mice. In the WT mice, AKAP5 anchors both PKA and CaN to PSD95 in proximity to AMPARs. There are other AKAPs and other CaN binding proteins associated with PSDs that can also bind PKA and CaN, respectively. In the AKAP5 KO PSD domains, the relative amounts of PKA and CaN are reduced and PKA redistributes to empty MAP2 binding sites in the dendritic shafts and to the cell body. In the D36 PSD's, because AKAP5D36 still binds CaN, there is an imbalance favoring increased phosphatase activity with PKA showing the same redistribution seen in the KO.

One hypothesis to explain the more severe electrophysiological and behavioral effects of the D36 mutant is illustrated in Fig. 8. AKAP5 binds both kinases (PKA and PKC) and a phosphatase (calcineurin) and we suggest that the kinase/phosphatase balance in the dendritic spine is critical in the regulation of downstream targets. Because the D36 mutant protein can target calcineurin but not PKA, it has the ability to act as a quasi dominant negative scaffold on targets that can be bidirectionally regulated, such as the GluR1 type of AMPAR. In the KO spines, neither the kinase nor the phosphatase can regulate targets through AKAP5 targeting. In D36 mutant spines, however, the AKAP targets the phosphatase without PKA and this imbalance might be responsible for the D36 dominant effects. Both PKA and calcineurin bind to AKAP5 and have been shown to be key regulators of GluR1 currents [57]. PKA and calcineurin act in opposition to each other to determine the relative GluR1 current by regulating trafficking to and from the plasma membrane as well as affecting single channel properties. In the complete AKAP5 KO both PKA and calcineurin would be released from their anchored sites in proximity to the AMPAR and we postulate that the mice with this congenital defect are able to partially compensate although that compensation does not appear to entail movement of a substantial fraction of PKA back to the normal dendritic sites.

Interestingly, GluR1 mutant mice with targeted mutations in the sites for PKA (S845A) and CaMKII (S831A), display a partial defect in hippocampal LTP and a complete deficit in LTD [52]. These GluR1 mutants exhibit normal Morris Water Maze learning and memory but display more subtle deficits in spatial learning when animals are required to learn and remember a new platform location [52], [54]. The electrophysiological and behavioral phenotypes of the GluR1 (S845A/S831A) mutants are quite similar to the phenotypes we observed for the AKAP5 D36 mutants, and is consistent with results showing that inhibitors of all PKA/AKAP interactions can disrupt synaptic plasticity and certain forms of spatial memories [58].

AKAP5 scaffolded molecules such as PKC, PKA, and calcineurin are able to act on GluR1, and we have previously shown that forskolin induced GluR1 phosphorylation is reduced in the D36 mice [23]. However, the hypothesis that the D36 phenotypes might be due solely to dysregulation of AMPAR phosphorylation is an oversimplification, as there are numerous other PKA and calcineurin substrates near the PSD that can affect synaptic plasticity, including stargazin, NR1 subunits of the NMDAR, β-ARs, and L-type calcium channels [12], [59], [60], [61]. Additionally, it is worth noting that the cGMP dependent kinase, PKGII, can phosphorylate GluR1 at Ser 845 and increase surface expression of GluR1, downstream of NMDAR activation [62]. Thus there are parallel mechanisms acting on GluR1, which affect synaptic plasticity. However, the GluR1 pools that act as PKA and PKG substrates might be different, as deleting the SAP97 interacting domain on GluR1 abrogates PKA, but not PKG phosphorylation of GluR1 at Ser845 [62]. It has recently been shown that AMPAR internalization also requires AKAP5-PSD95 and AKAP5-calcineurin interactions [63]. Together, these results demonstrate that bidirectional AMPAR regulation by PKA and CaN requires targeting of these two proteins via AKAP5 interactions within the PSD domain. Our unexpected result demonstrating the gain of function effects in the D36 mutant compared to the KO underscores the complexity of analyzing mouse mutants of the AKAP scaffolding proteins since they assemble with multiple signaling partners that may interact with each other as well as potential substrates.

During the course of our investigation, Tunquist et al generated an AKAP5 KO and reported alterations in PKA localization, hippocampal LTD, AMPAR rundown, M-current suppression, and several behavior tasks [64]. Our studies on the AKAP5 KO agree with those of Tunquist et al on the delocalization of PKA and the apparent lack of substantial AKAP compensation but differ in the analysis of LTD and behavior. The role of PKA and other signaling pathways in hippocampal electrophysiology changes during early development [23]. Our LTD studies were done with very young (10–14 day-old) mice whereas the Tunquist study was done with adults and this may contribute to differences. Clearly the KO mice do have phenotypes and we have demonstrated neuronal, vascular, and cardiac abnormalities. The KO mice display deficits in neuronal L-type calcium channel phosphorylation in response to β-agonists [12], defective regulation of vascular smooth muscle by angiotensin II through a PKC dependent pathway [65], and loss of β-adrenergic enhanced calcium-induced calcium release in ventricular cardiomyocytes (B. Nichols and GSM, unpublished). Taking our studies together with those of Tunquist et al [64], we suggest that both the KO and D36 exhibit electrophysiological and behavior deficits that are dependent on animal age, the specific behavioral task employed, and perhaps other parameters such as genetic background.

In a recent paper, the localization of type II PKA was analyzed at high resolution in dendritic shafts and spines using both cultured neurons and an in vivo approach with transfected PKA subunits [66]. The authors conclude that MAP2 is the major type II PKA binding AKAP in neurons and localizes the kinase to the dendritic shafts and excludes PKA from spines. Our results and those of Tunquist et al are not consistent with this recent report [64]. It is clear that MAP2 is an important dendritic AKAP that plays a substantial role in type II PKA localization in dendrites [39] and MAP2 mutant mice have decreased dendritic PKA [67]. An amino-terminal deletion mutant of MAP2 that removes the PKA binding site, leads to PKA delocalization from dendrites as well as behavioral effects [68]. However, these MAP2 knockout and mutant mice also have striking deficits in dendritic elongation and dendritic architecture [69], [70] that could contribute to the mislocalization of PKA and perhaps the mislocalization of other scaffolding proteins such as AKAP5.

Our results show a dramatic delocalization of type II PKA subunits in the AKAP5 knockout and D36 mice. Since MAP2 mutants also affect PKA localization, we conclude that MAP2 and AKAP5 are probably working in concert to localize PKA in dendrites. One intriguing possibility comes from our observation that a fraction of AKAP5 is associated with MAP2 suggesting that MAP2 might be binding and facilitating the microtubule-dependent transport of synaptic components to the dendritic spines.

In this study, we describe phenotypic responses that underscore the importance of the balance between the opposing activities of kinases and phosphatases at the post-synaptic terminals. AKAP5 is likely to play a key role in mediating this bidirectional plasticity that occurs both in response to electrical stimulation paradigms [71] and in vivo following learning [72]. The AKAP5 KO and D36 mice described here provide a valuable tool to dissect the role of this AKAP in dendritic localization of PKA and other AKAP5 binding partners and together with other mutant AKAP mice may help unravel the complex spatial signaling that forms the substrate for synaptic plasticity.

## Supporting Information

Figure S1 Relocalization of RIIβ subunits in the cell body area and dendritic shaft in KO CA1 pyramidal neurons. Immunohistochemistry of RIIβ at high power in pyramidal CA1 neurons. RIIβ staining is shown at the cell body and proximal apical dendritic region in WT vs. KO neurons
(1.24 MB TIF)

Figure S2 SC/CA1 LTP and LTD from AKAP5 KO hippocampal slices are normal. A, Summary traces before (light traces) and 60 minutes after (dark traces) LTP induction. B, Summary traces in WT vs. KO mice before (light traces) and 60 minutes after (dark traces) the LTD induction protocol.
(2.25 MB TIF)

Figure S3 Normal motor learning and drug induced motor response in AKAP5 KO and D36 mice. A, Motor learning and memory was measured by repeated trials on an accelerating rotarod over two consecutive days in AKAP5 KO and D36 mice compared to their WT littermate controls. Day 1 trials assess learning of the task, and day 2 trials assess memory for the task over the 24-hour period as well as peak performance on the rotarod. ANOVA analysis shows significant effect of day 1 trials for KO group: F (5,132) = 38.1, p<0.001, and for D36 group: F (5,132) = 27.2, p<0.001 but no genotype effect on day 1 trials for KO (p = .71) or D36 (p = .64) mice. There was no significant effect on day 2 for trial or for genotype. B, Cataleptic motor response to a haloperidol challenge in KO and D36 mice compared to their WT littermate controls. Responders are mice that maintain a semi-upright posture for 20 seconds when placed in that position by the experimenter. Haloperidol dose was 4 mg/kg delivered by IP injection, and motor behavior was assessed 15 minutes, 1, 3, and 7 hours after IP injection.
(1.80 MB TIF)
