# Supplementary figures and images for: Mutations in AKAP5 Disrupt Dendritic Signaling Complexes and Lead to Electrophysiological and Behavioral Phenotypes in Mice
Source: PLoS One. 2010 Apr 23;5(4):e10325. doi: 10.1371/journal.pone.0010325 (PMC2859064; doi:10.1371/journal.pone.0010325)

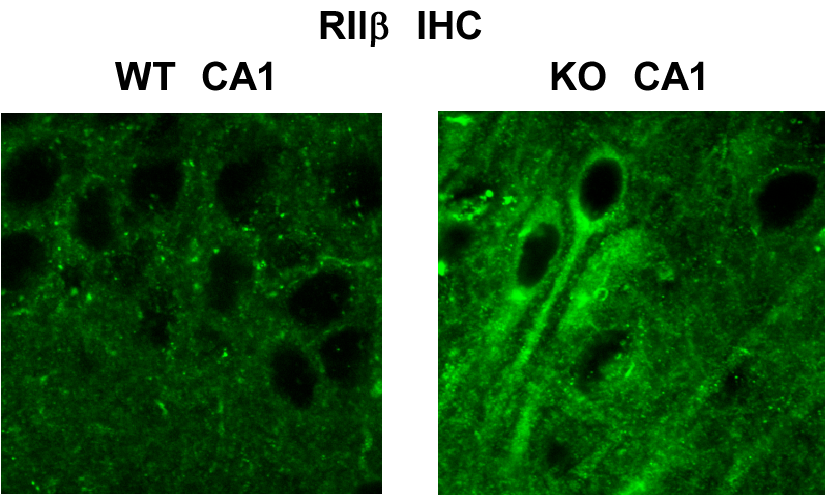

Supplement: Figure S1 — Relocalization of RIIβ subunits in the cell body area and dendritic shaft in KO CA1 pyramidal neurons. Immunohistochemistry of RIIβ at high power in pyramidal CA1 neurons. RIIβ staining is shown at the cell body and proximal apical dendritic region in WT vs. KO neurons (1.24 MB TIF) [file pone.0010325.s001.tif]

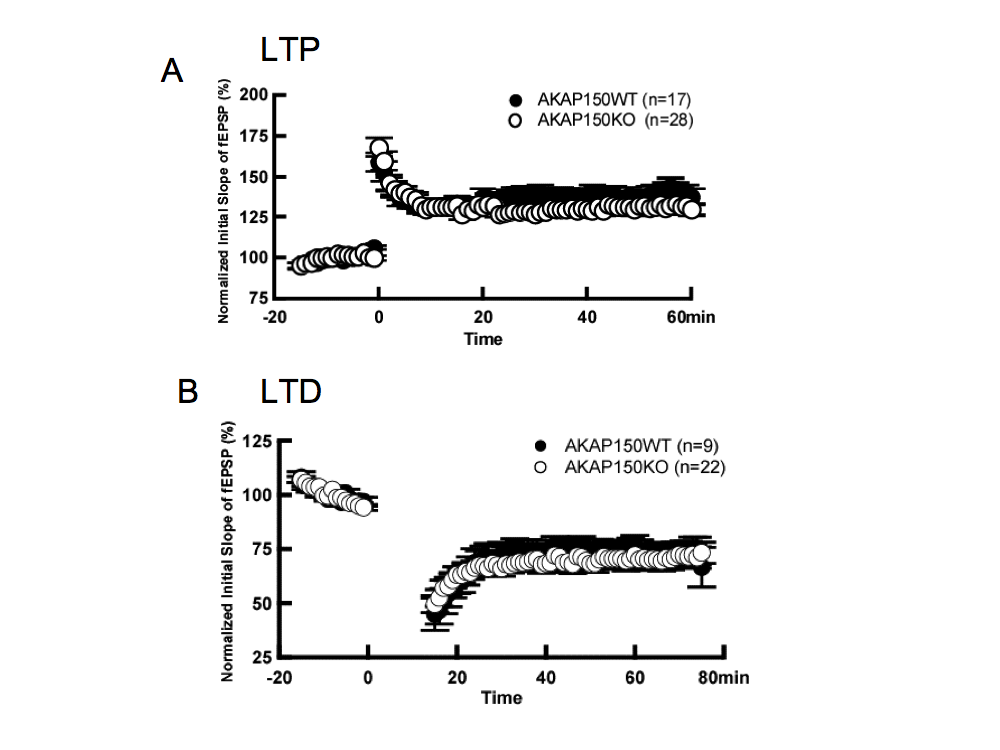

Supplement: Figure S2 — SC/CA1 LTP and LTD from AKAP5 KO hippocampal slices are normal. A, Summary traces before (light traces) and 60 minutes after (dark traces) LTP induction. B, Summary traces in WT vs. KO mice before (light traces) and 60 minutes after (dark traces) the LTD induction protocol. (2.25 MB TIF) [file pone.0010325.s002.tif]

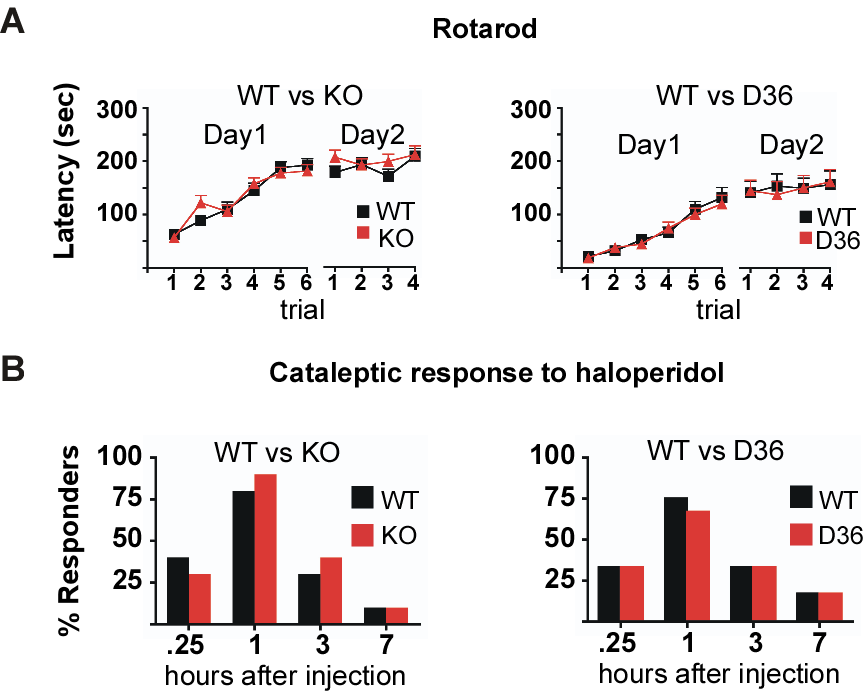

Supplement: Figure S3 — Normal motor learning and drug induced motor response in AKAP5 KO and D36 mice. A, Motor learning and memory was measured by repeated trials on an accelerating rotarod over two consecutive days in AKAP5 KO and D36 mice compared to their WT littermate controls. Day 1 trials assess learning of the task, and day 2 trials assess memory for the task over the 24-hour period as well as peak performance on the rotarod. ANOVA analysis shows significant effect of day 1 trials for KO group: F (5,132) = 38.1, p<0.001, and for D36 group: F (5,132) = 27.2, p<0.001 but no genotype effect on day 1 trials for KO (p = .71) or D36 (p = .64) mice. There was no significant effect on day 2 for trial or for genotype. B, Cataleptic motor response to a haloperidol challenge in KO and D36 mice compared to their WT littermate controls. Responders are mice that maintain a semi-upright posture for 20 seconds when placed in that position by the experimenter. Haloperidol dose was 4 mg/kg delivered by IP injection, and motor behavior was assessed 15 minutes, 1, 3, and 7 hours after IP injection. (1.80 MB TIF) [file pone.0010325.s003.tif]
